# The mortality rate and years of life lost due to cancer in a City of North of Islamic Republic of Iran during the years 2013–2021: joinpoint regression analysis

**DOI:** 10.1097/MS9.0000000000003055

**Published:** 2025-03-05

**Authors:** Habibollah Azarbakhsh, Hossein-Ali Nikbakht, Andishe Hamedi, Seyedeh Niko Hashemi, Pouyan Ebrahimi, Mohammad-Ali Jahani

**Affiliations:** a Ahvaz Jundishapur University of Medical Sciences, Ahvaz, Iran; b Social Determinants of Health Research Center, Health Research Institute, Babol University of Medical Sciences, Babol, Iran; c Student Research Committee, Shiraz University of Medical Sciences, Shiraz, Iran; d Doctorate of Medicine, Shiraz University of medical sciences, Shiraz, Iran; e Student Research Committee, Babol University of Medical Science, Babol, Iran

**Keywords:** age- standardized mortality, cancer, premature death, years of life lost

## Abstract

**Background:**

By providing a comprehensive analysis of incidence, mortality, and disability, cancer burden studies are tools for cancer control planning. The present study investigates the mortality rate and the years of life lost (YLL) due to cancers in a 9-year period.

**Methods:**

All registered cancer deaths in a city of north of Iran during 2013–2021 were considered. Crude, standardized mortality, and YLL of cancers were calculated for different age and sex groups. Also, to check the trend of the YLL, the joinpoint regression has been utilized.

**Results:**

During the study years (2013–2021), 3294 cancer deaths occurred. The total YLL because of cancer in the 9 years of the study were 22 228 (9 per 1000 people) in males, 20 824 (8.6 per 1000 people) in females, and 43 052 (8.8 per 1000 people) in both sexes. The average YLL due to malignancy was 11.7 years in males and 15.0 years in females. The highest percentage of YLL in men was caused by cancers of digestive organs and respiratory organs, and in women, cancers of digestive organs and breast.

**Conclusion:**

The YLL because of cancer were increasing in both men and women. Addressing the prevention of the underlying causes of YLL – especially gastrointestinal and breast cancers – may significantly affect overall life expectancy.

**HIGHLIGHTS**

- An estimated 250 million DALY (disability-adjusted life years) are due to malignancies.
- Among the 22 groups of illnesses and injuries in the GBD 2019 study, cancer is second only to heart problems in terms of mortality, and DALY in the world.
- Cancer in Iran has grown more than twice in terms of DALY from 1990 to 2016.
- Cancer burden is estimated using different indicators such as prevalence, incidence rate, mortality rate, and DALYs.

## Introduction

In recent years, the pattern of diseases in the world has changed towards non-communicable diseases, including cancer. The increase in urbanization, demographic changes of the population, and the progress of health care have been the main factors of this change^[1]^. Almost three-quarters of mortality due to non-communicable diseases, including cancers, takes place in countries with low- and middle-income such as Iran. This issue has resulted in the health systems of these countries facing a double burden of disease^[2]^. Cancers have been introduced as the first or second cause of death in many countries in 2020^[3]^. According to data released by the International Agency for Research on Cancer affiliated with the World Health Organization (GLOBOCAN IARC2021), in 2020, more than 19 million new cases of malignancy and nearly 10 million deaths from it took place in the world in all ages and both genders^[4]^. An estimated 250 million DALY (disability-adjusted life years) are due to malignancies. Among the 22 groups of illnesses and injuries in the GBD (global burden of disease) 2019 study, cancer is second only to heart problems in terms of mortality, YLL (years of life lost), and DALY in the world^[5]^.

Cancer burden studies facilitate the development of specific policies and programs by comprehensively analyzing incidence, mortality, and disability for all cancers. These studies are tools to support policymakers and health managers in making better decisions^[6]^. Cancer burden is estimated using different indicators such as prevalence, incidence rate, mortality rate, and DALYs^[7]^. DALY is a key tool in evaluating the burden of cancer because it combines YLL due to early death and years lived with disability (YLD) caused by a specific disease. This index measures both morbidity and mortality by quantifying the disease burden^[8]^. Globally, the DALYs index has increased by 16% from 2010 to 2019^[5]^. Cancer in Iran has grown more than twice in terms of DALY from 1990 to 2016^[9]^. Also, premature death from cancer has increased from 420 to 530 thousand people per year between 2011 and 2016 respectively. This increase was mainly due to an increase in cancer in adulthood and aging, specifically among males^[10]^. Cancers are known to be a major public health problem in Iran, accounting for more than one million and three hundred thousand YLLs^[11]^. Therefore, it is necessary to promote prevention and control programs and policies at the national level, especially in the cities, to improve health indicators.

Policymakers use cancer burden studies to plan the national cancer control program. Most of the studies conducted in the region and Iran have examined the burden of cancer through mortality, incidence, and prevalence, and the studies that have evaluated the YLL index at the regional or national level are minimal. Considering the epidemiological changes of cancer burden indicators in Iran and the world and with regards to the importance and the effect of these diseases on people’s lives, we conducted this research to investigate the mortality rate and the YLL due to cancer during a 9-year period.

## Methods

The population studied in this research was all the registered cancer deaths of City during 2013–2021 in the System of Registration and Classification of Causes of Death of the Health Department of University of Medical Sciences, and the sampling method was a census. The System of Registration and Classification of Causes of Death in the Health Department obtains its data from valid death certificates from various sources, such as mortuaries, forensic medicine, hospitals, and all doctors trained in the registration of the causes of death who obtain special booklets for registering the causes of death from Health department. We extracted all deaths from cancer from the population-based Electronic Death Registration System (EDRS) by age, sex, and year of death and based on ICD-11 (International Classification of Diseases). The codes used in this study were C00-D48. In the population-based electronic death registration system, all available sources were used to detect, record, and collect information about death, then these deaths are sent to the Health Vice-Chancellor of the University of Medical Sciences.

Also, since one of the most important steps in the analysis of the mortality causes is to check the quality of the information on the causes of death data, codes of the cause of death were reviewed and modified as ill-defined or null when appropriate. We controlled the data in regards to recording duplicate cases, controlling the variables of the data with other recorded information, the codes of the causes of death improbable in terms of gender and age, and improbable cause of death codes in terms of lethality. The causes of death, which were impossible or very rare in terms of sex and age, were requested from the relevant place and corrected by the relevant experts by referring to the relevant records. After repeated sampling, the redistribution of absurd and ill-defined codes was done. After approval by the program officials in the Ministry of Health, it was suitable for citation and final reporting. The work has been reported in line with the STROCSS criteria^[12]^ (Supplementary Digital Content, available at: http://links.lww.com/MS9/A743)

Death data from 2013 to 2021 were extracted from the country’s death registration system software. The death causes were coded based on the 11th edition of the International Classification of Diseases and Mortality (ICD-11). The death data report was in Excel file format, so the required fields, including gender, type of cancer, and age, were filtered in Excel, and then the coding of the mentioned fields was done, and the data was transferred to SPSS.

## Statistical analysis

Crude and standardized mortality rates^[5]^ of cancer were calculated based on sex and year of death in the study period. To determine YLL, the calculation was performed by means of the standard life table and determining the life expectancy for various age and gender groups, in addition to the number of mortalities because of cancer, in every age and gender group, and according to the following relationship^[13,14]^:

$$YLL=NCe{\;}^{\left(ra\right)}/{\left(\beta +r\right)}^{2}\left[e^{-\left(\beta +r\right)\left(L+a\right)}\left[-\left(\beta +r\right)\left(L+a\right)-1\right]-e^{-\left(\beta +r\right)a}\;\left[-\left(\beta +r\right)a-1\right]\right],$$

where *N* is the number of people who died at a specific age and sex; *L* is the standard life expectancy of the deceased at the same age and gender; *r* is the discounting rate that equals 0.03; *β* is the contractual rate in determining the age value and equals 0.04; *C* is the adjusted fixed value, which is equal to 0.1658; *a* is the age of death; and *e* is constant and equals 2.71.

The analysis of the number of YLL due to premature death due to cancer was done utilizing the YLL template of 2015, World Health Organization in Excel version 2016. First, the YLL was calculated according to 18 age groups: 0–4, 5–9, 10–14, etc., up to 85 years old, and then based on age groups 0–4, 5–14, 15–29, 30–44, 45–59, 60–69, 70–79 and over 80 years are shown in a figure. In order to determine the trend of crude and standardized mortality rates and the number of YLL for different years, the joinpoint regression has been utilized. Joinpoint regression analysis illustrates the pattern of change in successive segments of time and the decrease or increase in every segment^[15,16]^.

The resulting line segment between joinpoints is described by the annual percent change (APC) according to the slope of the line segment and the average annual percent change (AAPC). The analysis for the trend was carried out utilizing the Joinpoint Regression software 4.9.1.0.

## Results

During the 9-year study period (2013–2021), 3294 cancer mortality happened in City. Of these, 57.9% (1906 cases) were in men. As seen in Table 1, the crude mortality rate because of cancer in males increased from 58.2 (per hundred thousand population) in 2013 to 84.8 per hundred thousand population in 2021 (*P* for trend = 0.021). In women, it increased from 35.4 (per hundred thousand population) in 2013 to 70.3 (per hundred thousand population) in 2021 (*P* for trend = 0.005). The standardized mortality rate in men increased from 49.6 per hundred thousand in 2013 to 69.8 per hundred thousand in 2021 (*P* for trend = 0.030). In females, it grew from 7.30 30.7 per hundred thousand population in 2013 to 57.4 per hundred thousand in 2021 (*P* for trend = 0.013) (Table 1).

**Table 1:** Crude and standardized mortality rate (per 100 000 population) and years of life lost because of cancer by gender and year between 2013 and 2021

Year	No. death		Crude mortality rate		ASR (95%CI)		YLL			
							No.		(Per 1000)	
	Male	Female	Male	Female	Male	Female	Male	Female	Male	Female
2013	149	90	58.2	35.4	49.6 (40.3–59.0)	30.7 (23.4–38.0)	1776	1428	6.9	5.6
2014	147	114	56.6	44.3	46.3 (37.2–55.5)	39.7 (31.6–47.8)	1618	1640	6.2	6.4
2015	162	116	61.4	44.5	51.3 (41.9–60.8)	39.0 (30.9–47.1)	2023	1785	7.7	6.8
2016	238	171	88.9	64.7	74.8 (63.5–86.1)	52.9 (43.2–62.6)	2725	2580	10.2	9.8
2017	227	159	83.1	59.1	66.2 (55.4–77.0)	49.5 (40.3–58.6)	2626	2358	9.6	8.8
2018	256	183	91.9	66.8	71.8 (60.6–83.1)	55.7 (46.0–65.4)	2826	2745	10.1	10.0
2019	253	188	89.1	67.4	74.8 (63.8–85.8)	54.1 (44.4–63.7)	3043	2766	10.7	9.9
2020	224	164	77.4	57.8	61.8 (51.7–72.0)	44.8 (35.9–53.6)	2537	2396	8.8	8.4
2021	250	203	84.8	70.3	69.8 (59.3–80.3)	57.4 (47.7–67.1)	3054	3126	10.4	10.8
Total	1906	1388	77.3	57.1	63.5 (60.0–66.9)	47.5 (44.5–50.5)	22 228	20 824	9.0	8.6
*P* value	–	–	0.021	0.005	0.030	0.013	–	–	0.015	0.005

**Figure 1.**
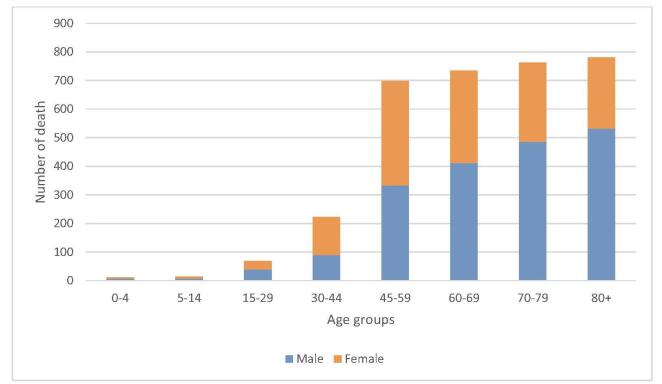
Rate of cancer mortality by sex and age groups.

The highest number of mortalities was in men above 80 years and women between 45 and 59 years, and the smallest number of deaths in both sexes was in patients under 5 years (Fig. 1).

### Years of life lost because of cancer

The overall YLL because of cancer throughout the 9 years of our study were 22 228 (9 per 1000 people) in males, 20 824 (8.6 per 1000 people) in females, and 43 052 (8.8 per 1000 people) in both sexes (male/female sex ratio, 1:1) (Table 1). The average number of YLL because of cancer was 11.7 years in males and 15.0 years in females.

**Figure 2.**
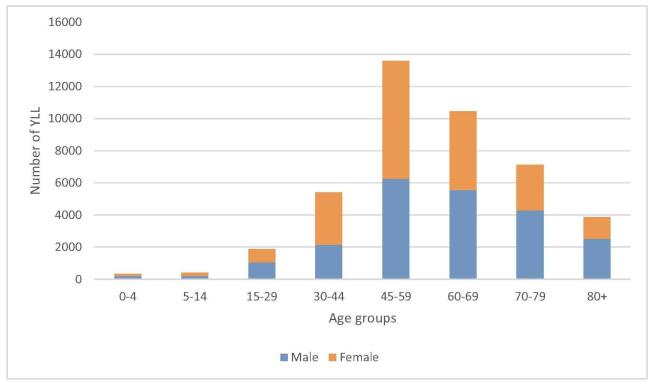
Years of life lost because of cancer by sex and age groups.

The highest number of YLLs in both sexes was between the ages of 45 and 59, and the lowest was in men aged 5 to 14 and women under 5 (Fig. 2).

As shown in Table 2, the highest percentage of lost years of life in men are caused by cancers of the digestive organs, respiratory and intrathoracic organs, and malignancies of the hematopoietic structures and related tissue. In women, it is due to cancer of digestive organs, breast cancer, and malignant neoplasms of female reproductive organs (Table 2).

**Table 2:** Years of life lost because of cancer according to gender and type of cancer between the years 2013–2021

Cancer type	Number of deaths			Number of YLL			% Total YLL	
	Male	Female	Total	Male	Female	Total	Male	Female
Malignancies of the lip, oral cavity, and pharynx	15	18	33	165	253	418	0.7	1.2
Malignant neoplasms of digestive organs	832	513	1345	9412	6567	15 980	42.3	31.5
Cancers of respiratory and intrathoracic organs	277	118	395	3285	1630	4915	14.8	7.8
Cancers of bone and articular cartilage	14	11	25	244	162	406	1.1	0.8
Cancers of skin, mesothelial, and soft tissue	24	15	39	320	211	531	1.4	1.0
Malignant neoplasm of breast	1	255	256	6	4570	4577	0.0	21.9
Cancers of female genital organs	0	124	124	0	2080	2080	0.0	10.0
Cancers of male genital organs	223	0	223	1734	0	1734	7.8	0.0
Cancers of urinary tract	63	21	84	682	275	957	3.1	1.3
Cancers of eye, brain and other segments of central nervous system	125	116	241	2024	1912	3936	9.1	9.2
Cancers of thyroid and other endocrine tissues	12	16	28	156	238	394	0.7	1.1
Cancers of poorly defined, secondary and not specified sites	52	42	94	642	635	1277	2.9	3.0
Cancers of lymphoid, hematopoietic and related tissue	233	124	357	3126	2058	5184	14.1	9.9
In situ neoplasms, Benign cancers and malignancies of uncertain or unknown behavior	35	15	50	432	233	665	1.9	1.1
Total	1906	1388	3294	22 228	20 824	43 052	100.0	100.0

**Figure 3.**
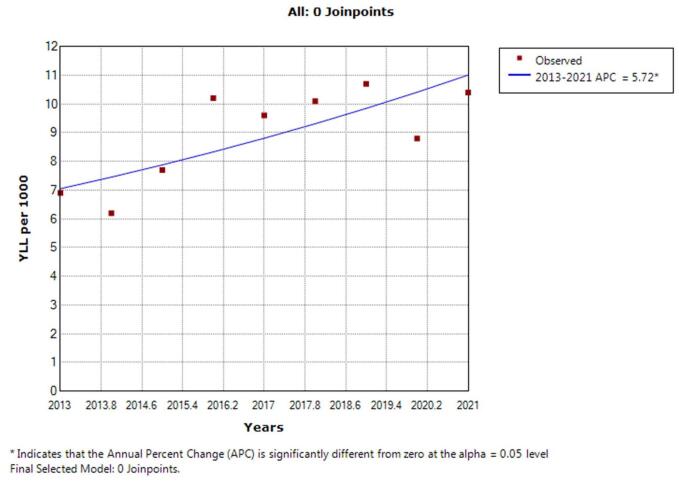
Patterns in the number of years of life lost because of cancer in men between the years.

**Figure 4.**
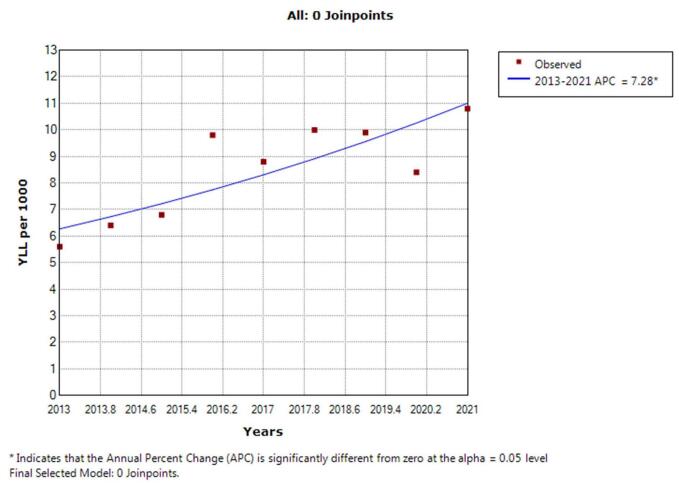
The pattern of years of life lost due to cancer in females between 2013 and 2021.

According to the joinpoint regression analysis, the 9-year trend of YLL rate because of cancer was increasing: the APC was 5.7% (95% CI 1.4 to 10.2, *P* = 0.015) for men, 7.3% (95% CI 2.9 to 11.9, *P* = 0.005) for women, and 1.9% (95% CI 0.8 to 3.1, *P* = 0.003) for both sexes. This model did not demonstrate any joinpoint; therefore, the AAPC is the same as the APC. (Figs. 3, 4).

## Discussion

The results of this research investigate the mortality rate and YLL due to cancer in a city in northern Iran from 2013 to 2021, providing insights into the burden of cancer on the population. The findings reveal significant increases in both crude and standardized mortality rates, as well as notable differences in YLL between genders and age groups.

The results of various studies also show that the incidence of cancer and its mortality differ in various times and regions and according to each region’s population characteristics^[17]^.

This study highlights an increase in cancer mortality rates. The crude mortality rate for men rose from 2.58 per 100 000 in 2013 to 8.84 per 100 000 by 2021, while women experienced an increase from 35.4 per 100 000 to 70.3 per 100 000. This trend is consistent with global observations where cancer mortality rates have been rising, particularly in low- and middle-income countries due to factors such as urbanization, lifestyle changes, and limited access to healthcare services^[5]^. Research performed in Brazil revealed that the age-standardized death rate was approximately 70 per 100 000 for men and 58 per 100 000 for women during the years 1980 to 2018^[18]^. There has also been a significant increase in the number of newly diagnosed cancer cases in Norway in the past 15 years, and cancer is the main reason for premature death in Norway^[19]^. In China, the age-standardized mortality rate (ASMR) increased steadily between 2000 and 2011^[20]^. A study in Iran in 2021 investigated the lost years of life due to cancer mortality during the years 2011 to 2018 and showed that an average of 43 000 people died due to cancer in Iran every year. During the study period, the death rate from cancer has been increasing, as in the present study^[10]^. In contrast, studies from high-income countries like the United States have reported declining ASMRs due to effective screening and early detection programs^[21]^. An analysis of cancer trends in the United States between 1998 and 2012 found that the ASMR was on a downward trend. England also began implementing population-based cancer screening to detect breast and cervical cancer in the 1980s. This measurement partially decreased breast cancer ASMR from 28.92 per 100 000 to 15.90 per 100 000 between 1990 and 2013^[17]^. This disparity reveals the need for improved cancer control strategies in Iran to address the increasing burden of cancer. Therefore, learning effective cancer prevention and management methods is of particular importance.

In the current study, the total YLL due to cancer was 43 052, with an average of 11.7 years for men and 15.0 years for women. The results of a research carried out by Pham in Japan like this study reported the average number of lost years of life because of cancer in men as 13.6 and in women as 17.5 years^[22]^. The higher average YLL in women aligns with findings from other studies indicating that women often experience greater YLL due to cancers that predominantly affect them, such as breast and reproductive organ cancers. The study’s observation that the highest number of YLLs occurred in individuals aged 45–59 is particularly relevant, as this age group is often affected by cancers that lead to significant premature mortality. In YLL as a measure of cancer burden on a national level study which conducted by Brustugun in 2012, from 25.8% of deaths and 35.2% of YLL caused by cancer among adults, a lower percentage of deaths occurred in women (28.7% in men and 23.1% in women), with A higher deficit of YLL occurred in women (32.8% in men and 37.8% in women). On average, women lost more years than men due to cancer (14.9 vs. 12.7 years), which is similar to the results of the present study^[19]^. In 2012, from 25.8% of deaths and 35.2% of YLL caused by cancer among adults, a lower percentage of deaths occurred in women (28.7% in men and 23.1% in women), with A higher deficit of YLL occurred in women (32.8% in men and 37.8% in women). On average, women lost more years than men due to cancer (14.9 vs. 12.7 years), which is similar to the results of the present study^[19]^. Also, a study that has been conducted to investigate the expected years of life loss due to adult cancer deaths in Yazd city (2004–2010), like the present study, shows that premature deaths due to cancer caused 40 753, YLL (5823 YLL annually). From 2004 to 2010, YLL from cancer as a fraction of total YLL increased from 12.8% to 15.2%, and women lost on average more years to cancer than men (11.6 in against 9.8 years)^[23]^. The gender differences observed in cancer mortality rates are significant. Men accounted for 57.9% of the total cancer deaths, men generally have higher cancer incidence and mortality rates compared to women. However, the finding that women lost more years of life on average due to cancer highlights the impact of specific cancers on life expectancy. Research indicates that while men may have higher overall cancer mortality rates, certain cancers disproportionately affect women during critical life stages, contributing to higher YLLs^[24]^. For instance, breast cancer is a leading cause of YLL among women aged 45–59, which corresponds with the findings from this study.

The results of the current study also revealed that the highest percentage of lost years of life in men are caused by cancers of the digestive organs, respiratory and intrathoracic organs, and malignancies of the hematopoietic lymphatic structure and related tissue, and in women, cancers of digestive organs, breast cancer and malignant neoplasms of female reproductive organs. The significant increase in YLL per death for cancers of the gastrointestinal tract is likely due to the increased mortality rate at a younger age for this type of cancer. The spectrum of cancer types is different from a developing country to a developed country. The different trends observed for the number of deaths and YLL can be attributed to increased population size, immigration, and life expectancy^[25]^. The highest number of deaths and YLL attributable to malignancy in the United States were from lung/bronchial, colon/rectal, breast, pancreatic, and liver cancers. Cancer burden estimates are not the same. Some cancers, such as prostate cancer, have a high mortality but have low YLL, while other cancers, such as testicular cancer, have a low mortality but have a high YLL. These differences are caused by the difference in age at the time of death. The effect of age on the burden of cancer plays a major role in calculating YLL per death^[26]^. This study found that the highest mortality rates were among men over 80 years and women aged 45–59 years, while the lowest rates were seen in children under 5. This pattern is consistent with global trends where older age groups are more susceptible to cancer-related deaths. Additionally, the low mortality rates in younger populations reflect the effectiveness of childhood health interventions but also point to a need for targeted prevention strategies for adult-onset cancers. Almost half (47%) of the world’s mortality due to cancer in 2020 was in people 70 years or older, and 41% of those who died were between 50 and 69 years old^[3]^. A study that was carried out in Iran in 2021 showed that the mortality rate attributable to cancer for men less than 30 years is 1.2 times that of women, and at the age of 50, the death rate of men is 1.5 times that of women. Between the ages of 30 and 70 years, the death rate of women is higher than that of men^[10]^, which can be caused by cancers that increase in women at this stage of life, including breast, uterus, and lung cancers. The peak incidence is for women between 40 and 60^[10,26]^. The results of this study also show that the highest percentage of lost years of life in women was related to cancers of the digestive organs, breast cancer and malignant neoplasms of the reproductive organs, which can be the reason for the higher age of death in women of 40–59 years old. In Japan, the highest burden of disease in men was esophageal, gastric, colorectal, and liver cancer, with a median YLL greater than 14 years, and the lowest for prostate cancer because it affected relatively older men, whereas breast cancer affected younger women. It showed that they died 28.5 years earlier on average which is in line with the results of the present study that the most common cancer in women was breast cancer and women had the most years of lost life^[22]^. According to GLOBOCAN 2020 estimates, most cancer-related mortalities and DALYs in China were related to gastrointestinal malignancies (colorectal, gastric, hepatic, and esophageal cancer). Lack of awareness of early screening leads to low rates of early cancer diagnosis, thereby affecting the outcome and DALY burden of patients living with cancer in China^[17]^.

The increasing trends in both mortality rates and YLL due to cancer underscore an urgent need for enhanced public health initiatives focused on prevention, early detection, and treatment of cancers prevalent in this region. Furthermore, implementing comprehensive screening programs similar to those seen in higher-income countries could significantly impact early detection and treatment success. The effectiveness of cancer screening is majorly dependent on population coverage and how the target population adheres to the program^[27]^. Chronic infection prevention is known to be the most helpful strategy for preventing infection-related malignancies such as gastric, liver, cervical, and nasopharyngeal cancers. Helicobacter pylori (H. pylori) infection is the main risk factor for malignancies of the stomach^[28]^. Tobacco use is one of the major contributing factors for different cancers around the world, which is related to about 20 different cancers, and more than 70% of respiratory system cancer cases in the world are due to tobacco use. Hence, the implementation of strict smoking control policies is a valuable measure to decrease the incidence and age-standardized mortality in this cancer^[29]^. Improving knowledge about a healthy lifestyle through education is another way to prevent cancer. Unhealthy lifestyles such as high BMI, consumption of red meat in high quantities, and inactivity are some of the causes of the increase in lung, breast, colorectal, and prostate cancer^[30]^.

One of the limitations of this research was that YLL was not compared in Iran as a whole because of the lack of availability of the needed data. One of the strengths of the current study is the use of population-based data on all registered cancer deaths in City to estimate cancer-related mortality and YLL over 9 years from 2013 to 2021. The broad time range and suitable sample size are other strengths of this study.

## Conclusion

Cancer death rates have increased from 2013 to 2021. The present study suggests that a more accurate and comprehensive calculation of mortality statistics would be useful in relation to research funding and discussion of public health issues. Cancer is a major and increasing cause of premature death, and YLL may be a more accurate measure of the number of deaths. These data may be useful for developing regulatory strategies and funding priorities.

According to the findings of this study, the trend of raw and standardized mortality rates, as well as the number of YLL due to cancer, has been increasing in both sexes. Also, the highest percentage of lost years of life in men was caused by cancers of digestive organs, respiratory organs, and intrathoracic organs, and in women, cancers of digestive organs and breast cancer. Therefore, addressing the prevention of the main causes of YLL – particularly gastrointestinal and breast cancers – may significantly impact overall life expectancy. More investigation is needed to determine the causes of cancer incidence and mortality.

## Supplementary Material

## Data Availability

The datasets used and/or analyzed during the current study are available from the corresponding author on reasonable request.

## Research registration unique identifying number (UIN)

Approval and registration of the proposal in the Babul University Research Council and then obtaining the ethics code number (IR.MUBABOL.HRI.REC.1402.167.)
